# Molecular Mechanisms Underlying Flower Bud Differentiation in *Osmanthus fragrans* Lour.

**DOI:** 10.3390/plants14233577

**Published:** 2025-11-23

**Authors:** Qinghua Yang, Min Zhang, Lin Chen, Xianrong Wang

**Affiliations:** Co-Innovation Center for Sustainable Forestry in Southern China, College of Life Sciences, Nanjing Forestry University, Nanjing 210037, China; yangqinghua204@163.com (Q.Y.); zhangmin@njfu.edu.cn (M.Z.); clinechen@njfu.edu.cn (L.C.)

**Keywords:** *Osmanthus fragrans*, flower bud differentiation, blooming phenology, molecular mechanisms

## Abstract

*Osmanthus fragrans*, an evergreen tree or shrub belonging to the Oleaceae family, is widely utilized in landscaping, food processing, and the fragrance industry for its unique aroma and ornamental values. Through natural and artificial selection, *O. fragrans* has diverged into two horticultural groups: the Asiaticus Group, which blooms year-round, and the Autumn Flowering Group, characterized by concentrated flowering in autumn. This phenotypic diversity is paramount importance for enhancing landscape value, extending the harvest periods, and meeting commercial demands. However, current research on flowering period differences among *O. fragrans* cultivars primarily focuses on physiological traits such as flower bud differentiation and phenological traits, with limited studies at the genetic and molecular levels. This article summarized the research progress in the classification characteristics, flower bud differentiation stages and processes, and the molecular mechanisms of flowering in *O. fragrans*, with a particular emphasis on the key genes that influence environmental factors such as high temperature, low temperature, and drought on the flowering period, and the regulatory mechanisms underlying the repeated flowering of the Asiaticus Group. The aim is to provide a theoretical foundation for breeding new cultivars with varied flowering times. Future research on *O. fragrans* will employ multi-omics technologies to systematically elucidate the key genes, signaling pathways, and epigenetic networks that regulate flower bud differentiation. A primary objective is to elucidate the synergistic interactions between environmental factors and endogenous hormones, thereby establishing precise models for flowering regulation and guiding practical production applications of *O. fragrans*.

## 1. Introduction

*Osmanthus fragrans*, commonly referred to as sweet *Osmanthus*, is a plant classified within the genus *Osmanthus* of the Oleaceae family. It is one of Chinese top ten traditionally famous flowers, valued for its edible, medicinal, and ornamental properties, leading to its extensive use across various applications. *O. fragrans* embodies significant traditional Chinese cultural values, and has a long history of cultivation. Historical records indicate that the cultivation can be traced back to the Spring and Autumn and Warring States periods (ca. 3rd century BCE). Throughout the history of Chinese literature, it has been frequently celebrated in both poetry and prose, establishing itself as a distinctive cultural symbol. In the contemporary era, it embodies friendship, honor, and auspiciousness, with its cultural significance permeating areas such as cultivation, landscaping, and folk traditions. In medicinal applications, the Dictionary of Chinese Medicine records that its roots, branches, flowers, and fruits can be utilized as herbal remedies to dispel dampness and cold, resolve phlegm, and disperse blood stasis. It has been employed to alleviate conditions such as wheezing and cough induced by phlegm, as well as intestinal wind and dysentery [1]. In culinary contexts, the flowers of *O. fragrans* have a long history as an ingredient in food products. Items such as *Osmanthus* tea and cake are widely appreciated for their distinctive flavors. These flowers contain various secondary metabolites, and their acetone extract exhibits antioxidant and preservative properties that contribute to delaying skin aging. In landscaping applications, different cultivars of *O. fragrans* are esteemed for their elegant form, rich fragrance, and auspicious symbolism, making them common choices in horticultural design [2].

**Figure 1:**
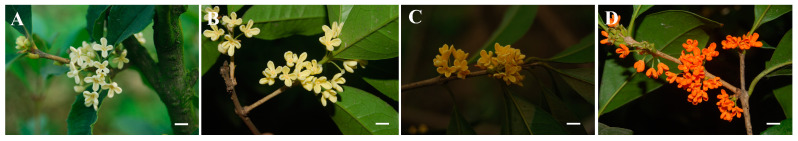
Four cultivar groups of *O. fragrans*, (**A**) Asiatiacus Group; (**B**) Albus Group; (**C**) Luteus Group; (**D**) Aurantiacus Group. Scale bar = 5 mm.

With a cultivation history of over 2500 years in China, *O. fragrans* is widely cultivated in the regions of south of the Qin-ling Mountains–Huai River, where it has established five principal production areas: Xianning city in Hubei Province, Chengdu city in Sichuan Province, Hangzhou city in Zhejiang Province, Suzhou city in Jiangsu Province, and Guilin in Guangxi Province. Furthermore, it has been designated as the city flower in 26 cities throughout China [3,4]. Throughout prolonged cultivation, *O. fragrans* has developed numerous intraspecific variations due to artificial selection, natural hybridization, and environmental factors, resulting in the formation or breeding of many cultivars. Cultivated varieties of *O. fragrans* can be classified into four cultivar groups based on distinct characteristics such as flowering season, inflorescence type, and flower color (Figure 1). The primary classification criterion is the flowering season, which divides the specimens into the Autumn Flowering Group and the Asiaticus Group. The secondary criterion is flower color, which further categorizes them into the Albus Group, Luteus Group, and Aurantiacus Group, thus establishing a total of four cultivar groups [5,6].

Nevertheless, the molecular mechanisms underlying the flowering differences between the Asiaticus Group and the Autumn Flowering Group remain inadequately understood. Current research on variations in floral phenology among *Osmanthus* cultivars have primarily focused on physiological aspects, including flower bud differentiation and blooming phenology. However, the precise regulatory mechanisms governing flower bud differentiation and blooming processes, along with their coordinated interactions with environmental factors, have yet to be thoroughly elucidated. This limited understanding has resulted in empirical approaches for horticultural interventions that lack both precision and broad applicability. Therefore, a more comprehension of the flowering mechanisms in *O. fragrans* to accurately regulate its flowering period can effectively extend its ornamental duration. This is crucial for enhancing the economic and social benefits associated with landscape aesthetics, ensuring a stable supply of raw materials for the *O. fragrans* industry, including flower harvesting and processing, and fostering advancements in cultivation techniques.

## 2. Phenology and Flower Bud Differentiation Characteristics of *O. fragrans*

Phenology is a discipline that studies the interrelationships between periodic variations in living organisms and environmental conditions. As a significant economic and ornamental plant species, research on the phenology of *O. fragrans* holds significant implications for guiding cultivation management practices, optimizing flowering periods, and enhancing economic benefits. The flowering period of *O. fragrans* is typically concentrated in autumn. Through phenological observations of various cultivars, the flowering process could generally be divided into 11 stages: flower bud emergent stage, flower bud bursting stage, ball-shaped stage, top bracts stage, bud-pedicel stage, bud-eye stage, primary blooming stage, pre-full blooming stage, full blooming stage, late-full blooming stage, and flower fading stage [7]. Phenological observations of various *O. fragrans* cultivars in Nanjing and Kaifeng revealed that the overall flowering period was notably brief, lasting about half a month from ball-shaped stage to flower fading stage, The optimal ornamental period was limited to merely 5–7 days [8].

Phenological observations of *O. fragrans* reveal cultivar and regional variations in its flowering process, and the meteorological conditions before flowering are the primary external factors regulating the flowering period. Using Nanjing as a case study, Principal Component Analysis (PCA) of the phenological data pertaining to the full-bloom period of *O. fragrans*, alongside meteorological records from 2000 to 2005 for the 1 to 5 weeks preceding flowering, indicates that the weekly average minimum temperature prior to blooming exerts the most significant influence on the flowering period. A continuous decline in this temperature during the four weeks leading up to flowering promotes flower bud initiation, with optimal conditions occurring when the temperature falls below 18 °C in the week immediately preceding flowering. Appropriate precipitation prior to flowering can enhance air humidity, thereby promoting flowering. However, excessive rainfall tends to delay the flowering period, while drastic fluctuations in temperature adversely affect the flowering process [9].

Flower bud differentiation is a critical transition phase in plants, marking the shift from vegetative to reproductive growth. It involves the transformation of leaf buds into flower buds, followed by the development of floral organs, representing a complex morphogenetic process [10]. Flower bud differentiation is a prerequisite for flowering, and studying its progression is essential for manipulating flowering time, improving flower yield, and enhancing quality [11,12,13]. Current research on the phenotypic aspects of flower bud differentiation in *O. fragrans* primarily relies on morphological anatomy, tissue sectioning, and microscopic observation. The growth and development of *Osmanthus* flower buds occur after the maturation of new shoots [14,15]. Studies on the flower bud differentiation process of ‘Jingui’ and ‘Houban Jingui’ indicated that timing and corresponding stages of flower bud differentiation as follows: bract differentiation commenced in mid-April, bract primordia emerged in early May, inflorescence primordia developed in late June, flower bud primordia appeared in early July, sepal primordia of the terminal flower were formed by mid-July, petal primordia progressed from mid to late July, stamen primordia and anthers differentiated between late July to late August, and carpel primordia were established by the end of August. The rate of flower bud differentiation demonstrated a slow-fast-slow pattern [16,17]. Although there were many cultivars of *O. fragrans* with significant differences in morphological and fertility, the stages of flower bud differentiation were generally similar across cultivars. These stages included initial differentiation, involucre differentiation, inflorescence primordium differentiation, top flower sepal differentiation, stamen differentiation and pistil differentiation stages.

**Figure 2:**
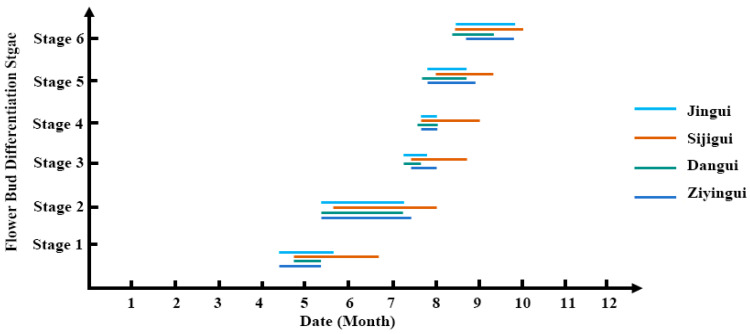
The processes of flower bud differentiation of in four *O. fragrans* cultivars [18]. Note: Stage 1, Initial differentiation stage; Stage 2, Involumcrum differentiation stage; Stage 3, Inflorescence primordium differentiation stage; Stage 4, Top flower sepal differentiation stage; Stage 5, Stamen differentiation stage; Stage 6, Pistil differentiation stage.

The Asiaticus Group is characterized by its capacity to produce flower multiple times throughout the year and the presence of peduncles, distinguishing it from the Autumn Flowering Group. Research on flower bud differentiation within the Asiaticus Group has been relatively limited. Microscopic observation of the flower bud differentiation process in ‘Ziyingui’ (Albus Group), ‘Dangui’ (Aurantiacus Group), ‘Jingui’ (Luteus Group), and ‘Sijigui’ (Asiaticus Group) revealed that the initiation of flower bud differentiation occurred at a similar time across the four cultivars, but the duration varied among them. Each stage of flower bud differentiation persisted for a longer period in the Asiaticus cultivar compared to the Autumn flowering cultivars. While differentiation had concluded in the Autumn Group, it continued in the Asiaticus Group, aligning with its trait of multiple, sequential flowering events (Figure 2) [18]. Research on the flower bud differentiation of ‘Tianxiang Taige’ revealed three different types of flower buds: normal flower buds, special flower buds, and leaf-like flower buds (termed “taige” flower buds). The morphological differentiation of normal and special flower buds was similar, with both having pistils degenerated into leaf-like carpels. The proliferate-flower bud produced atrophied pistils, which were replaced by meristems that ultimately differentiated into leaves and branches [19,20].

Research on flower bud differentiation of the Asiaticus Group predominantly focuses on the autumn season, with even fewer studies addressing the flower bud differentiation related to its flowering in other seasons. The main flowering differences between the Asiaticus Group and Autumn Flowering Group were as follows: (1) the Asiaticus Group bloomed three times a year, while Autumn Flowering Group flowered predominantly in autumn; (2) the Asiaticus Group underwent three distinct cycles of flower bud differentiation annually, each with varying durations—approximately two months starting in March, two and a half months starting in June, and one month starting in October; (3) during spring and winter, the inflorescences of Asiaticus Group developed at the apex and leaf axils of current-year shoots as panicles, with winter blooms exhibiting peduncles. In contrast, the Autumn Flowering Group produced axillary cymes without peduncles on current-year shoots [21,22].

## 3. Molecular Regulatory Mechanisms of Flower Bud Differentiation and Flowering in *O. fragrans*

### 3.1. Regulation of Flower Bud Differentiation and Flowering by Environmental Factors

#### 3.1.1. Impact of Low Temperature on Flower Bud Differentiation

##### Negative Regulation of Flowering Repressors by Low Temperature Signals

Low temperature serves as a critical environmental signal for flower bud differentiation in plants, facilitating the flowering process through the regulation of various gene expressions. The *SVP* (*SHORT VEGETATIVE PHASE*) gene family, functioning as MADs-box transcription factors, typically played either inhibitory or promotional roles in the temporal regulation of flower development. Studies have shown that low-temperature signals in *O. fragrans* can promote flower bud differentiation by regulating the expression of the *OfSVP4*, *OfSVP6*, and *OfSVP7* genes. Under low-temperature conditions, the expression patterns of these *OfSVP* genes change, which may regulate downstream flower development-related genes such as *FT* (*FLOWERING LOCUS T*) and *SOC1* (*SUPPRESSOR OF OVEREXPRESSION OF CONSTANS1*), thereby releasing the inhibitory state of flower bud differentiation and promoting the formation and development of floral buds [23]. Further studies revealed that under relatively low temperature conditions (10–15 °C), *OfSVP6* suppressed GA biosynthesis and signal transduction by interacting with the *OfDELLA* protein in the GA signaling pathway or by regulating genes such as *OfGA20ox*, and subsequently synergized with the ABA pathway to ultimately delay flowering in *O. fragrans* [24].

##### Positive Regulation of Flowering Promoters by Low Temperature Signals

Low temperature stimulation is essential for promoting flower bud development and sustaining flowering in *O. fragrans*. Several gene families, such as *OfSPL* (*SQUAMOSA PROMOTER-BINDING PROTEIN-LIKE*), *OfFCA* (*FLOWERING LOCUS CA*), and *OfTCP* (*TEOSINTE BRANCHED 1/CYCLOIDEA/PROLIFERATING CELL*), have been identified as participating in the pathway of temperature-regulated flower bud differentiation.

*miR156* is a significant microRNA that typically delays plant developmental processes and flowering time by negatively regulating members of the *SPL (SQUAMOSA PROMOTER BINDING PROTEIN-LIKE)* gene family [25]. In *O. fragrans*, environmental low temperature significantly inhibited the expression of *miR156*, thereby relieving its transcriptional repression on the target genes *OfSPL6* and *OfSPL10*, and leading to the upregulation of these two genes. Meanwhile, *OfSPL1A*, *OfSPL5*, and *OfSPL13* are also involved in growth and development under low temperature conditions, but not regulated by *miR156* [26].

*FCA* is a crucial gene family that regulates self-fertilization in plants, encodes proteins containing RNA-binding domains, and participates in post-transcriptional regulatory processes that influence flowering time [27]. In *O. fragrans* ‘Yanhonggui’, the expression level of *OfFCA* gene under low-temperature conditions (19 °C) was significantly higher than at ambient temperature (25 °C), indicating that this gene could sensitively respond to environmental temperature changes, actively participate in flower bud differentiation, and consequently promote earlier flowering time [28].

*TCP* family genes are essential for plant growth, development, and stress responses. Whole-genome analysis of *O. fragrans* revealed that Class I *TCP* genes were involved in organ morphogenesis by regulating cell proliferation and differentiation [29]. Under different temperature treatments (19 and 25 °C), *OfTCP5*, *OfTCP9* and *OfTCP12* might serve as key genes responsive to low temperatures in promoting flower bud differentiation in ‘Yanhonggui’. However, the response of *OfTCP9* to low temperatures was relatively delayed, with its primary function occurring during the later stages of flower bud differentiation. Furthermore, an analysis of transcriptional activation activity indicated that neither *OfTCP5* nor *OfTCP12* possessed transcriptional activation activity [30].

Low temperature also induced genome-wide DNA hypomethylation in *O. fragrans*, particularly characterized by overall reductions in methylation levels across CG, CHG, and CHH sequences. This process was mediated through activation of demethylases such as *OfROS1a*, which specifically triggered genomic hypomethylation (particularly within CHH sequence), thereby achieving dual regulatory functions: modulating the auxin signaling pathway to promote peduncle elongation, or regulating expression of genes associated with sugar and lipid metabolism as well as temperature response. The dynamic equilibrium between methyltransferases and demethylases maintained methylation homeostasis, collectively facilitating floral induction in *O. fragrans* [31].

##### The Effect of Low Temperature on the Petal Formation

Low temperature not only affects flowering gene expression, but also significantly influences structural genes involved in the flowering process. In *O. fragrans*, the size of adaxial and abaxial epidermal cells in petals at different developmental stages gradually increased during flower opening, suggesting that cell expansion in petals was a major factor in flower opening [24]. XTH (XYLOGLUCAN ENDOTRANSGLU-COSYLASE/HYDROLASES) were cell wall-loosening proteins involved in cell wall relaxation, leading to petal cell enlargement. Among these, a total of 38 *OfXTH* genes were identified in the *O. fragrans* genome, with *OfXTH24*, *OfXTH27*, *OfXTH32*, *OfXTH35*, and *OfXTH36* potentially responding to environmental temperature changes to regulate flower opening [32]. Further, in the *O. fragrans* ‘Yanhonggui’, *bHLH79* (*BASIC/HELIX-LOOP-HELIX* *79*) was found to directly regulate the high expression of *OfXTH28* to control petal cell enlargement [33].

#### 3.1.2. High Temperature Signals Inhibit Flowering in *O. fragrans*

In contrast to the promotion of flowering by low temperature, high temperature inhibits flower bud differentiation in *O. fragrans*. The key gene, *OfC3H49*, was proved to involve in environment temperature-mediated floral transition through *C3H* gene family identification and transcriptome sequencing under different temperature treatments in *O. fragrans* ‘Sijigui’ [34]. The results showed that high temperature directly activated the promoter activity of *OfC3H49*, and *OfC3H49* was regulated by *OfWRKY17*, inhibiting the floral transition in *O. fragrans* in response to high ambient temperature. *OfC3H49* could directly bind to the promoter of *OfSOC1B* and inhibited its transcription, while also suppressing the expression of downstream flowering promoters such as *OfFT*, *OfAP1a* (*APETALA1 a*), *OfLFY* (*LEAFY*), and *OfSOC1B* [35].

#### 3.1.3. Impact of Drought on Flowering

In addition to temperature, drought conditions also affect flowering in *O. fragrans*. The plasma membrane intrinsic protein gene *OfPIP2* exhibited significant up-regulation during the flowering period under drought conditions. *OfPIP2* increased petal size by regulating epidermal cell size, and the transcription factor *OfMYB28* directly regulated the expression of *OfPIP2*, thereby enhancing the drought tolerance of *O. fragrans* [36].

### 3.2. Regulatory Mechanisms of Multi-Season Flowering

Previous research on the flowering of the Asiaticus Group primarily focused on phenological observations and morphological studies. With advancements in molecular biology and sequencing technologies, researchers have commenced the exploration of the regulatory mechanisms and molecular genetic basis underlying its multi-season flowering. Flowering-related genes such as *TFL1* (*TERMINAL FLOWER 1*), *SOC1* (*SUPPRESSOR OF OVEREXPRESSION OF CO 1*), *AP1* and *BFT* have been identified as critical roles.

The flowering genes *FT* and its homologs *TFL1*, *SOC1*, and *AP1* were cloned from the Asiaticus Group ‘Fodingzhu’ and the Autumn Flowering Group ‘Yanhonggui’, and their functions were investigated. The results showed that *OfTFL1A* had very low expression in autumn but significantly increased during the differentiation from calyx to petals in winter in ‘Fodingzhu’. In contrast, *OfTFL1B* was not expressed during winter floral transition but showed high expression during autumn differentiation. This suggested that the two *TFL1* genes in *O. fragrans* may have distinct roles. The differential expression patterns of *OfFT* and its homolog *OfTFL1* in the Asiaticus Group during autumn and winter may be one of the factors contributing to the varied flowering mechanisms across seasons. Heterologous expression of *OfFT*, *OfSOC1A*, and *OfSOC1B* in *Arabidopsis* resulted in early flowering phenotypes, further indicating that these genes promote flowering [37].

Transcriptome comparison in the Autumn Flowering Group and the Asiaticus Group showed that *OfAP1* and *OfTFL1* were identified as candidate genes responsible for multi-season flowering in the Asiaticus Group [38]. Heterologous overexpression in *Arabidopsis* demonstrated that *OfAP1-b1* and *OfTFL1-1* promote early flowering, whereas *OfAP1-b2* and *OfTFL1-2* delay flowering. The different alternative splicing transcripts have distinct effects on flowering time, indicating functional divergence [39,40]. Recently, *OfAP1-a* was discovered to be regulated by the *LFY* homolog *OfLFY* in the Asiaticus Group ‘Sijigui’. Yeast one-hybrid and dual-luciferase assays confirmed that *OfLFY* inhibits *OfAP1-a* expression by directly binding to its promoter. Conversely, *OfAP1-a* can also bind to the *OfLFY* promoter region and promote its expression. Overexpression of *OfAP1-a* in *Arabidopsis* not only accelerated flowering but also altered petal number [41].

Additionally, three floral integrators, *OfFT* (*FLOWERING LOCUS T*), *OfTFL1*, and *OfBFT*, were revealed significant differences during the floral transition between single-flowering and continuous-flowering varieties of *O. fragrans* transcriptome analysis. *OfFT* acts as a flowering activator, while *OfBFT* functions as a flowering inhibitor. The gene *OfSPL8* was identified as a common upstream transcription factor for both, suggesting its important role in regulating continuous flowering [42]. Besides repressing flowering, the *OfBFT* gene also inhibits the formation of proliferating flowers in *O. fragrans*. Two distinct floral morphologies, normal flowers and proliferating flowers, coexisted in ‘Tianxiang Taige’. Transcriptome sequencing at different developmental stages of these two flower types revealed significant differential expression patterns of the PEBP family genes *OfBFT-a* and *OfBFT-b*, which showed continuous down-regulation during proliferating flower development. *OfBFT-a* and *OfBFT-b* directly bond to the promoters of the C-class gene *OfAG-a* and E-class gene *OfSEP3-a* within the ABC/DE model, suppressing their expression to regulate carpel development and proliferating flower formation. This provides new insights into the mechanisms of proliferating flower development and floral form breeding in flowering plants [43].

### 3.3. Molecular Regulation of Flower Senescence in O. fragrans

*O. fragrans* was an ethylene-sensitive flowering plant, and the senescence of its flowers was regulated by ethylene response transcription factors (*OfERFs*). Ten *OfAP2/ERFs* genes were potentially associated with floral senescence. It was discovered that *OfERF017* could accelerate flower senescence in *O. fragrans* by activating the catabolic pathway of organic acids, thereby reducing organic acid levels [44]. Furthermore, *OfACO2* (1-*AMINOCYCLOPROPANE-1-CARBOXYLIC ACID OXIDASE 2*) was identified as a key synthase gene responsive to ethylene regulation in *O. fragrans*. Its expression was up-regulated during flower senescence, induced by ethephon (which promotes ethylene synthesis), and suppressed by silver nitrate treatment (which inhibits ethylene action), indicating its involvement in ethylene-mediated petal senescence [45].

### 3.4. Regulatory Mechanisms Influencing Floral Organ Size

Current research on the molecular mechanisms underlying petal size variation in *O. fragrans* remains limited. In large-flowered cultivars (e.g., ‘Jinqiu Gui’, ‘Dahua Jingui’), the expression level of the GATA transcription factor family member *OfGATA9* was significantly higher compared to small-flowered cultivars (e.g., ‘Yu Linglong’, ‘Xiaoye Sugui’). Transgenic analysis demonstrated that *OfGATA9* positively regulated floral organ size through promoting cell expansion, providing a theoretical basis for breeding large-flowered *O. fragrans* cultivars [46].

DELLA proteins negatively regulate gibberellin (GA) signal transduction and influence plant growth and developmental expansion through cellular regulation. In *O. fragrans* ‘Tianxiang Taige’, ‘Sijigui’, ‘Tiannu Sanhua’ and ‘Rixiang Gui’, a negative correlation was observed between the expression level of the DELLA protein *OfRGA* (Regulate GA) and petal size. Cultivars with larger petals exhibited lower *OfRGA* expression levels, suggesting that *OfRGA* might inhibit petal growth. As a negative regulator in the GA signaling pathway, *OfRGA* suppressed cell expansion by down-regulating the expression of cell expansion-related genes, thereby modulating floral organ size. This mechanism provided a theoretical basis for flower-type breeding and molecular studies on floral organ development in *O. fragrans* [47].

## 4. Conclusions and Prospect

*O. fragrans* has diverged into two horticultural groups (the Autumn Flowering Group and Asiaticus Group) through long-term cultivation and evolution. This natural speciation with distinct flowering habits provides an ideal model for investigating flower bud differentiation in woody plants. In this article, we summarized the molecular mechanisms focusing on flower phenology, bud differentiation, and key regulatory genes. Physiologically, flower phenology encompasses 11 phases and bud differentiation involves 6 stages, with an extremely short flowering period (≈15 days) and optimal ornamental window (5–7 days). Genetically, confirmed flowering regulators include *OfSVP*, *OfTCP*, *OfSPL*, *OfFCA*, and the *OfWRKY17-OfC3H49* module, with *OfCCCH* [48] and *OfABF* [49] as potential candidates. These findings converge on a core “low temperature-induced *SVP*/*TCP*/*SPL*/*FCA*-*FT*/*SOC1*” regulatory model; however, upstream signal perception, cross-pathway regulation, and cultivar-specific mechanisms require further investigation.

Future research should integrate multi-omics approaches and gene editing to unravel the flowering regulatory network, providing a theoretical basis for precise flowering time control and molecular breeding in *O. fragrans*. Key priorities include: (1) completing genome sequencing of additional cultivars beyond the currently available ‘Rixiang Gui’ and ‘Liuye Jingui’ to advance understanding of *Osmanthus* origin and evolution; (2) expanding beyond dominant transcriptomic studies to comprehensive multi-omics integration for clarifying agronomic trait mechanisms; (3) establishing efficient stable genetic transformation systems to enable native functional validation, which is more compelling than heterologous validation in model plants; and (4) intensively investigating the molecular regulation of key ornamental and stress-resistant traits.

## Data Availability

No new data were created in this study.
